# Foreign Body in the Rectum

**Published:** 1845-07

**Authors:** 


*Foreign Body in the Rectum.*—Mr. Blanchard, of Pimlico, in a communication which he has obligingly forwarded us, states that he was called to a man whom he found in a state of great prostration, and apparently dying; on recovering from which the man complained of agonizing pain about the anus, with which he was seized while at the water-closet, where he fainted. Mr. Blanchard examined him, and saw a piece of black thread about an inch long hanging from the bowel. On introducing the finger, he found a needle with its point downwards about three inches up the rectum sticking in the gut. He experienced considerable difficulty in extracting it, in consequence of its position, &c., but after repeated and careful attempts, succeeded, and found it to be a tailor's needle with about half a foot of black thread attached. Inflammation commenced in the neighboring parts on the second day, but by the application of leeches and other antiphlogistic means, the man got well in a week.—*Ibid.*

